# Supplementary material for: Modification of Vip3Ab1 C-Terminus Confers Broadened Plant Protection from Lepidopteran Pests
Source: Toxins (Basel). 2019 Jun 3;11(6):316. doi: 10.3390/toxins11060316 (PMC6628392; doi:10.3390/toxins11060316)
Supplement: Supplementary file 1 [file toxins-11-00316-s001.pdf]

# Supplementary Materials: Modification of Vip3Ab1 C-Terminus Confers Broadened Plant Protection from Lepidopteran Pests

**Megan S. Sopko *, Kenneth E. Narva, Andrew J. Bowling, Heather E. Pence, James J. Hasler, Theodore J. Letherer, Cory M. Larsen and Marc D. Zack ***

**Table S1.** PCR primer sequences used to generate Vip3Ab1-740.

| Primer Name | |
|---|---|
| Vip3Ab1 Part A Forward Primer | 5′ GGA TCC GAA GGA GAT ATA CAT ATG GCA AAC ATG AAC AAC ACC AAA CTG AAC GCG 3′ |
| Vip3Ab1 Part A Reverse Primer | 5′ TCC AGT ATT TTC ATC TTT CAA GTA GAT TGA CGC CTT 3′ |
| DIG740 Part B Forward Primer | 5′ AAG GCG TCA ATC TAC TTG AAA GAT GAA AAT ACT GGA 3′ |
| DIG740 Part B Reverse Primer | 5′ GGA TCC CTA TTT AAT AGA AAT GTT TTC 3′ |

**Table S2.** PCR primer sequences for Arabidopsis copy number analysis.

| Primer Name | |
|---|---|
| DSM2 forward Primer | 5′ CTTTACGCCTCCCTCTTCGA 3′ |
| DSM2 reverse Primer | 5′ CTGAGCAATGCCAGCATAGG 3′ |
| TafII forward Primer | 5′ GAGGATTAGGGTTTCAACGGAG 3′ |
| TafII reverse Primer | 5′ GAGAATTGAGCTGAGACGAGG 3′ |

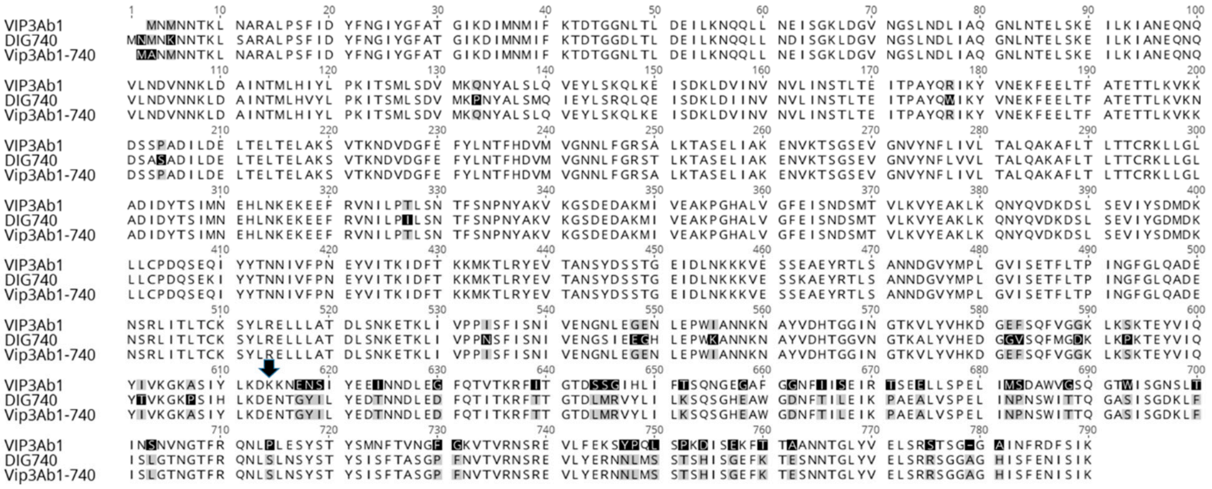

**Figure S1.** Sequence alignment of Vip3Ab1, DIG740 (Vip3Ai1) and Vip3Ab1-740. The black arrow denotes the location at which the chimera protein was generated. Black background shading is used to highlight amino acid diversity between proteins (BLOSUM62 substitution matrix).

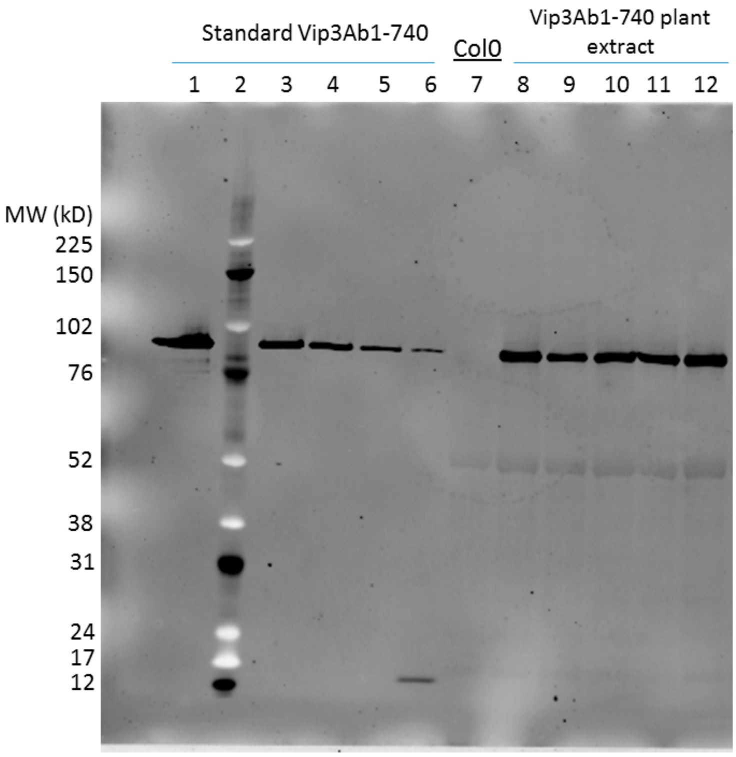


**Figure S2.** Representative Western Blot of Arabidopsis leaf extracts. “Col0” denotes Columbia-0 plant extract control to show background immunoreactivity of non-transformed Arabidopsis plants.

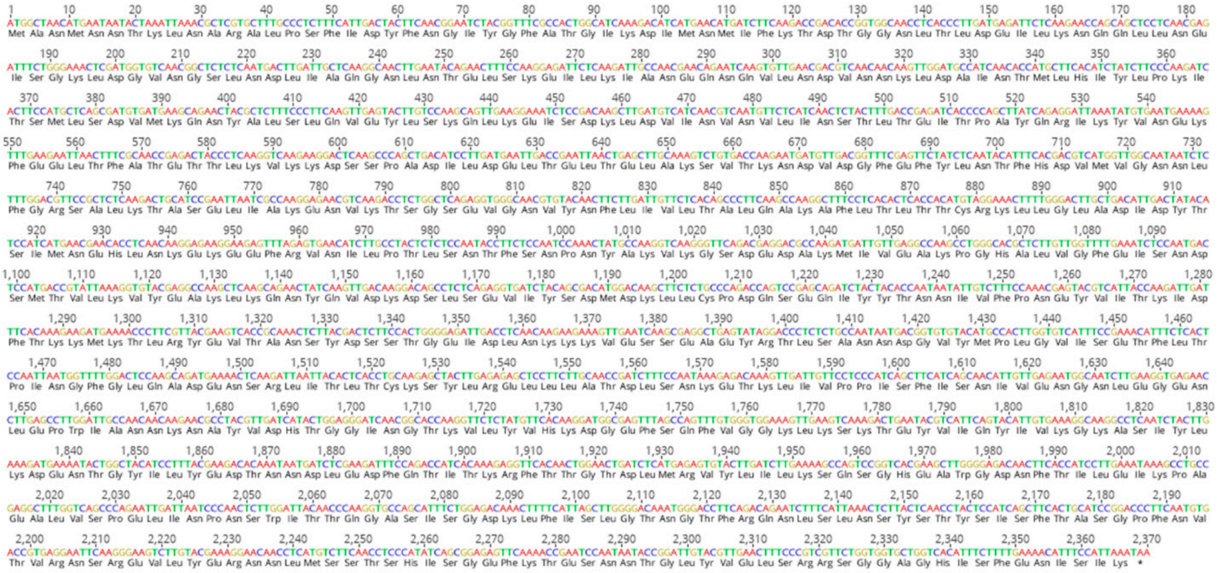

**Figure S3.** DNA sequence for Vip3Ab1-740 plant expression construct.
